# Ethanol treatment of nanoPGA/PCL composite scaffolds enhances human chondrocyte development in the cellular microenvironment of tissue-engineered auricle constructs

**DOI:** 10.1371/journal.pone.0253149

**Published:** 2021-07-09

**Authors:** Narihiko Hirano, Hirohisa Kusuhara, Yu Sueyoshi, Takeshi Teramura, Ananth Murthy, Shinichi Asamura, Noritaka Isogai, Robin DiFeo Jacquet, William J. Landis

**Affiliations:** 1 Department of Plastic and Reconstructive Surgery, Kindai University, Osakasayama, Japan; 2 Institute of Advanced Clinical Medicine, Kindai University, Osakasayama, Japan; 3 Division of Plastic and Reconstructive Surgery, Children’s Hospital Medical Center, Akron, Ohio, United States of America; 4 Department of Plastic and Reconstructive Surgery, Wakayama Medical College, Wakayama, Japan; 5 Department of Polymer Science, University of Akron, Akron, Ohio, United States of America; Ohio State University, UNITED STATES

## Abstract

A major obstacle for tissue engineering ear-shaped cartilage is poorly developed tissue comprising cell-scaffold constructs. To address this issue, bioresorbable scaffolds of poly-ε-caprolactone (PCL) and polyglycolic acid nanofibers (nanoPGA) were evaluated using an ethanol treatment step before auricular chondrocyte scaffold seeding, an approach considered to enhance scaffold hydrophilicity and cartilage regeneration. Auricular chondrocytes were isolated from canine ears and human surgical samples discarded during otoplasty, including microtia reconstruction. Canine chondrocytes were seeded onto PCL and nanoPGA sheets either with or without ethanol treatment to examine cellular adhesion in vitro. Human chondrocytes were seeded onto three-dimensional bioresorbable composite scaffolds (PCL with surface coverage of nanoPGA) either with or without ethanol treatment and then implanted into athymic mice for 10 and 20 weeks. On construct retrieval, scanning electron microscopy showed canine auricular chondrocytes seeded onto ethanol-treated scaffolds in vitro developed extended cell processes contacting scaffold surfaces, a result suggesting cell-scaffold adhesion and a favorable microenvironment compared to the same cells with limited processes over untreated scaffolds. Adhesion of canine chondrocytes was statistically significantly greater (p ≤ 0.05) for ethanol-treated compared to untreated scaffold sheets. After implantation for 10 weeks, constructs of human auricular chondrocytes seeded onto ethanol-treated scaffolds were covered with glossy cartilage while constructs consisting of the same cells seeded onto untreated scaffolds revealed sparse connective tissue and cartilage regeneration. Following 10 weeks of implantation, RT-qPCR analyses of chondrocytes grown on ethanol-treated scaffolds showed greater expression levels for several cartilage-related genes compared to cells developed on untreated scaffolds with statistically significantly increased SRY-box transcription factor 5 (*SOX5*) and decreased interleukin-1α (inflammation-related) expression levels (p ≤ 0.05). Ethanol treatment of scaffolds led to increased cartilage production for 20- compared to 10-week constructs. While hydrophilicity of scaffolds was not assessed directly in the present findings, a possible factor supporting the summary data is that hydrophilicity may be enhanced for ethanol-treated nanoPGA/PCL scaffolds, an effect leading to improvement of chondrocyte adhesion, the cellular microenvironment and cartilage regeneration in tissue-engineered auricle constructs.

## Introduction

The basic concept of tissue engineering proposed by Langer and Vacanti and colleagues [1, 2] was to integrate living tissue with biodegradable materials to achieve regeneration of natural structures in the body. In 1997, Cao and associates [3] advanced this idea by inducing regeneration of human ear-shaped cartilage and demonstrating that tissue engineering had the potential for auricular plastic surgery applications. Subsequent developments in auricle reconstruction have been made through several basic science investigations, in which animal and human chondrocytes have been utilized to design engineered auricle constructs [4–6]. Related studies have also shown that normal (conchal) and microtia chondrocytes from otoplasty are effectively equivalent for engineered auricular cartilage regeneration [7–9]. Additional advances in auricle fabrication by means of preclinical studies have been reported, demonstrating satisfactory human auricle tissue-engineered grafts [10]. On the other hand, such grafts required long-term chondrocyte expansion in vitro [10], and indeed, preclinical work has met with significant limitation. In a porcine autologous transplantation model, for example, the auricular implants were resorbed because of an inflammatory reaction which caused cartilage regression [11].

Success of auricular and other tissue regeneration requires careful consideration of numerous factors. These include cell phenotype [12–14], cell seeding density [15], selection of a scaffold appropriate to cells in its chemical and physical character [16], possible complement of growth factors for supporting cell proliferation and matrix production [17–19], the biomechanical nature of implanted structures [20, 21], and cell and tissue affinity for scaffold properties [22, 23]. Together, such aspects should provide a favorable, biocompatible microenvironment necessary for cell differentiation, proliferation, and matrix development leading to the formation of tissue-engineered constructs maintaining biological, biochemical, physical and biomechanical features, including three-dimensional (3D) structural size and shape, that faithfully mimic their natural tissue counterparts [24, 25].

Recent studies have shown that scaffold materials with a microstructure replicating the natural extracellular cartilage matrix hold promise for improving regeneration of this tissue [26–28]. In such a context, this laboratory has investigated cartilage regeneration by combining nanofibers of a biodegradable, principally hydrophilic polymer, polyglycolic acid (PGA), with poly-ε-caprolactone (PCL), a slowly bioresorbable, principally hydrophobic polymer having both human tissue biocompatibility and a component of mechanical strength, to produce a human ear-shaped biodegradable composite scaffold [29, 30]. The adhesion of seeded chondrocytes to nanofiber PGA (nanoPGA) is markedly improved compared to other previous biodegradable polymers, and favorable cartilage regeneration has been achieved in a large animal (dog) autologous transplantation model [31]. However, the thin, fragile nature of nanofibers in nanoPGA and similar nanopolymers has diminished their initial enthusiasm for clinical applications because of their inability to preserve the complex 3D shape and flexibility of a normal auricle.

The study reported here concerns a potential advancement in the development of a scaffold that supports cell structure and function in tissue engineering. To maintain a 3D auricle shape for extended times, the scaffold use of nanoPGA combined with PCL designed with optimal rigidity has been examined. In this instance, it was conceived that the surface of an ear-shaped PCL scaffold would be covered with nanoPGA to create a bioresorbable composite scaffold (nanoPGA/PCL). From a report that it could alter permeability and diffusion in polymer membranes [32], ethanol was also investigated as a possible agent to modify the nature, perhaps the hydrophilicity, of scaffold materials and thereby enhance cell-scaffold adhesion and the microenvironment for cell growth. As an initial set of experiments in vitro to explore this concept, canine auricular chondrocytes were seeded onto ethanol-treated individual sheets of PCL and nanoPGA and their adherence was compared to the same cells seeded onto identical scaffold sheets without ethanol treatment. Resulting data indicated ethanol treatment of each scaffold was favorable to increasing cell adhesion. Based on these findings, a subsequent group of experiments was conducted in which human auricular chondrocytes were seeded onto the novel nanoPGA/PCL composite scaffold designed as a small 3D-shaped human auricle. These tissue-engineered cell/scaffold constructs were exposed to ethanol or left untreated before chondrocyte seeding and were then subcutaneously implanted for 10 and 20 weeks in athymic (nude, *nu/nu*) mice. Putative induction of cartilage regeneration during implantation in vivo was documented by histology, histomorphometry and quantitative gene expression analysis.

It was found that the initial complex auricular spatial structure of the nanoPGA/PCL scaffolds was maintained and cartilage regeneration increased with implantation time up to 20 weeks. Constructs comprised of ethanol-treated compared to untreated scaffolds after 10 weeks of implantation in vivo yielded several interesting results. First, constructs with untreated scaffolds showed loss of chondrocytes and proteoglycans over this time period. Second, constructs with scaffolds treated with ethanol generated increases in certain cartilage-related gene expression levels and a correlative greater cartilage matrix production at the periphery and within these constructs. Additionally, after 10 weeks of implantation, inflammation- and apoptotic-related gene expression changed in the two types of constructs. Constructs comprised of ethanol-treated scaffolds compared to untreated scaffolds showed a statistically significant decrease in interleukin-1α (*IL1A*) expression and differences that were not statistically significant in caspase-8 (*CASP8*) or caspase-9 (*CASP9*). These results suggested that loss of chondrocytes over 10 weeks of implantation was a consequence of necrosis rather than apoptosis in constructs consisting of scaffolds that were not treated with ethanol. Moreover, during the same implantation time, ethanol treatment of nanoPGA/PCL scaffolds comprising constructs appeared to blunt the known inflammatory response of chondrocytes to polymers such as PGA and PCL [33–35] while promoting chondrocyte growth and development, and that effect continued with increasing cartilage regeneration over 20 weeks of construct implantation. These summary data support the use of nanoPGA/PCL scaffolds for extended maintenance of an auricle-shaped template and the application of ethanol on such composite scaffolds to enhance the cellular microenvironment of tissue-engineered constructs for improved regeneration of cartilage.

## Materials and methods

### Ethics statement

A protocol for obtaining normally discarded auricular cartilage remnants was approved by an Institutional Review Board at the Children’s Hospital Medical Center of Akron (CHMCA, Akron, OH) and samples were collected with parental-signed consent. Animal care including surgical and post-surgical monitoring was performed in compliance with the Institutional Animal Care and Use Committee (IACUC Protocol No. 17-07-163, Animal Welfare Assurance No. A3474-01) at the Northeast Ohio Medical University Comparative Medicine Unit (NEOMED CMU, Rootstown, OH) and according to the policies outlined by the National Institutes of Health Guide for the Care and Use of Laboratory Animals (NIH Publications No. 8023, revised 1978). Briefly, mice were observed weekly after surgery for signs of dehydration, lack of activity or presence of chronic wounds, and they were sacrificed if treatments implemented did not improve animal conditions within 24 hr. All canine experiments were performed with protocols and procedures conforming to the Protecting Human Research Participation and Guidelines for handling experimental animals (Protocol No. KAME-26-001, Registration No. 079, Kindai University Faculty of Medicine, Osaka, JP). The sole use of canine animals was for harvest and isolation of auricular chondrocytes.

### Preparation of polymer scaffolds

#### PCL sheets

A polymer solution containing 5% PCL (molecular weight: 270,000 Da) in 1,4-dioxane was gently poured into a template (1 cm x 1 cm cast) prepared beforehand and subsequently frozen at -40°C for 1 hr. The PCL polymer was then released from the template and freeze-dried at 40 Pa, -40°C for 12 hr (TF10-80ATA, Takara, Tokyo). Residual monomers and solvent were removed by vacuum drying (60°C, 12 hr) to complete the preparation of PCL sheets, each 1 cm x 1 cm x 2 mm in length, width, and thickness, respectively. PCL has known hydrophobicity [29].

#### NanoPGA sheets

NanoPGA was prepared using the melt blow method: Melted PGA was drawn into thin fibers by pushing it through multi-pores and the drawn fiber was dispersed using hot air to prepare non-woven nanoPGA sheets, 1 cm x 1 cm x 80 μm in length, width and thickness, respectively, comprised of polyglycolide with a mean fiber diameter of 1.2 μm [26]. For each nanoPGA sheet, volume density was 0.10–0.14 g/cm^3^ and porosity was 90.5–93.4% [26]. PGA has known hydrophilicity [30].

#### Ear-shaped nanoPGA/PCL composite scaffolds

Auricle-shaped 3D nanoPGA/PCL scaffolds were fabricated and provided by KLS-Martin Group (Muhlheim, Germany). Briefly, a bioresorbable porous core of PCL was designed and 3D-printed in the shape of the auricle from a young child. The helix to the lobe of the printed ear measured 3 cm, the tragus to the outer rim of the ear was 2 cm and the thickest part of the antihelix was 1 cm. The hydrophobic PCL frame was wrapped in a thin layer of hydrophilic nanoPGA (Gunze Co., Kyoto, JP) and secured by a simple melt technique (KLS-Martin Group). PCL then formed the inner portion of each resulting bioresorbable nanoPGA/PCL composite scaffold that was sterilized with ethylene oxide gas.

#### Ethanol treatment of PCL and nanoPGA sheets and nanoPGA/PCL composite scaffolds

As a means to examine the possible modification and improvement of the adherence of chondrocytes to the polymer examples prepared for this study, ethanol was utilized as a treatment preceding cell seeding of individual PCL and nanoPGA sheets as well as nanoPGA/PCL scaffolds. Polymer sheets or composite scaffolds were immersed in a sequentially decreasing regime of ethanol concentrations (100, 70, and 50%, 15 min each), then washed with sterile distilled water and phosphate buffered saline (PBS) for 15 min each. The sheets or composites were subsequently placed in DMEM/F-12 (Thermo Fisher Scientific Intl., Waltham, MA) containing 10% fetal bovine serum (FBS; Atlanta Biologicals Inc., Flowery Branch, GA) culture media for 60 min. Other individual polymer sheets or composite scaffolds processed identically as detailed above but without ethanol treatment were used as experimental controls in this study. All steps in the preparation of both ethanol-treated and untreated (control) sheets or scaffolds were conducted at room temperature (25°C) with the exception of DMEM/F-12 placement, which was carried out at 37°C.

### Experimental animals

Three normal female beagles, 12-24-weeks-old (purchased from Hamaguchi Laboratory Animals, Hyogo, Japan), were housed in individual cages under standard conditions at Kindai University (Osaka-sayama, Japan). Twenty male athymic (*nu/nu*) mice, 5-6-weeks-old (purchased from Envigo, Indianapolis, IN) were maintained in separate microisolation cages with sterile food and water in the NEOMED CMU (Rootstown, OH).

### Isolation of human and canine auricular chondrocytes

Auricular cartilage surgical remnants, typically discarded during treatment procedures, were obtained from adolescent patients undergoing either an otoplasty for prominent ears or an auricular reconstruction for microtia. Human auricular cartilage specimens were collected during surgery of prominent ears that were normal except for being markedly upright from the temporal head region because of an insufficient curvature of their antihelix. Including remnants from two microtia reconstruction surgeries, six cases were investigated of human auricular morphological abnormality (Table 1). The skin, subcutaneous tissue, muscle, and fat were removed aseptically from each normal (otoplasty) or microtia cartilage specimen. Cartilage remnants obtained from individual surgeries were kept separate and never mixed among cases. Cartilage remnants from each patient were subsequently dissected into 1 mm x 1 mm to 2 mm x 2 mm segments, and then they were treated with 0.3% collagenase type II (Worthington Biochemical, Freehold, NJ) at 37°C for 12 hr [36, 37]. After filtration through a nylon mesh with a 300-μm pore size, enzyme reactions were terminated by addition of complete medium comprised of DMEM/F-12 supplemented with 10% v/v FBS, 0.1% v/v gentamycin, 1% v/v penicillin/streptomycin (VWR Scientific Intl., Radnor, PA) and 0.1% v/v primocin (Invivogen, San Diego, CA). Cell suspensions were centrifuged at 4°C, 2,420 rpm for 8 min and then resuspended, and viable cells were counted by dye exclusion after staining with 0.4% trypan blue (VWR Scientific Intl.). Counted cells were seeded (1 x 10^4^ cells/cm^2^) in 75 cm^2^ cell culture flasks and cultured for one week at 37°C in 5% CO_2_ and 50% humidity. Changes in the complete medium were made every other day and included the addition of 0.25% L-ascorbic acid (VWR Scientific Intl.) and 10 ng/ml basic FGF (Kaken Pharmaceutical, Tokyo) [37]. Expanded cells from individual patients were then removed from flasks with 0.25% trypsin-EDTA solution (Thermo Fisher Scientific Intl.), pooled, recounted, passaged in 175 cm^2^ culture flasks for another week under the same conditions as above, then trypsinized, counted once more and used in the subsequent experiments as described below.

**Table 1:** Human surgical biopsy details for auricle regeneration.

Parameter	Mean ± SD
Patient age (yr)	5.6 **±** 2.4
Patient gender (male: female)	4: 2
Sample wet weight (g)	0.3 **±** 0.1
Cell yield per wet weight (x 10^6^/g)	9.5 **±** 2.1
Sample type (microtia: normal)	2: 4
Number of seeded and implanted constructs	20
Number of mice (initial: end point survival)	20: 17

Key: yr = year, g = gram, SD = standard deviation of mean value. Number of mice indicates those animals that were first implanted with a construct (20) and those animals whose constructs were retrieved for subsequent analysis following the end point of 10 or 20 weeks of implantation (17).

Auricular cartilage was harvested from three canines, processed and expanded in vitro following the same procedure detailed above for human biopsy cartilage. All canine surgeries were performed after confirmed death of the animals. Briefly, after a 12-hour fast, animals were sedated with xylazine (Selactar^®^, 0.15 mL/kg, Bayer Japan, Tokyo) injected intramuscularly and pentobarbital (Somnopentil^®^, 0.4 mL/kg, Kyoritsu Seiyaku, Tokyo) was given intravenously. The depth of sedation was monitored by eyelash reflex of the animals and additional pentobarbital was administered until death was confirmed with loss of this reflex. Both left and right canine auricles were dissected and auricular cartilage was obtained by removing the skin, cutaneous tissue, muscle, and perichondrium from the ears of the three dogs [38]. Chondrocytes harvested from left and right auricular cartilage of each dog (~ 10^7^ cells/ear) were pooled and the three pools of isolated cells were utilized separately for subsequent experiments.

### Seeding of canine auricular chondrocytes in vitro

Each of the three pools of canine chondrocytes from left and right auricles of the three individual dogs was randomly seeded at 1 x 10^5^ cells/cm^2^ on twelve individual PCL or twelve nanoPGA sheets placed in 6-well plates (Thermo Fisher Scientific Intl.) and maintained for 12 hr under 5% CO_2_ at 37°C. Half the number of PCL sheets (n = 6) and half the number of nanoPGA sheets (n = 6) were treated with ethanol as described above while the other half of these polymers were left untreated. After the culture period, all scaffolds were washed twice with PBS in preparation for scanning electron microscopy (SEM) and cell adherence analysis of chondrocyte numbers attached to the polymer sheets. PCL and nanoPGA sheets were intentionally over-seeded with canine chondrocytes at high concentration (1 x 10^5^ cells/cm^2^) at the outset of the experiment to assure that a maximal number of cells attached to these scaffolds. Cell attachment was determined by counting chondrocytes released from polymer sheets with 0.25% trypsin-EDTA solution (Thermo Fisher Scientific Intl.) following incubation for 10 min at 37°C and 5% CO_2_. Released cells were collected, concentrated and counted by the method of trypan-blue dye (Thermo Fisher Scientific Intl.) exclusion [37]. Cells from four of the six available polymer sheets from each of the four groups were counted twice and averaged.

### Scanning electron microscopy

The remaining two of six canine auricular chondrocyte-seeded polymer sheets from each of the four groups were fixed with 2.5% glutaraldehyde in 0.1 M Sorensen’s phosphate buffer at pH 7.4, washed with buffer (10 min, 6 x), and postfixed with 1% osmium tetroxide (Electron Microscopy Sciences, Hatfield, PA) for 60 min at 4°C followed by four buffer washes of 5 min each. Samples were then dehydrated in graded ethanols (50, 70, 80, 90, 95, and 100% at 4°C) and subsequently immersed briefly in t-butyl alcohol (3 x), frozen, and freeze-dried in a model VFD-20 freeze-drying apparatus (Vacuum Device Co., Ltd., Mito, Japan). The cell-seeded sheets were mounted with carbon paint (Nisshin EM Co., Ltd., Tokyo) on a small platform/stage, sputter-coated with 5 nm of platinum-palladium in a model E-102 ion sputter unit (Hitachi Ltd., Tokyo), and observed by SEM utilizing a model SU-3500 microscope operated at 5 kV and 139 μA (Hitachi Ltd.).

### Implantation in vivo of constructs comprised of human cell-seeded bioresorbable composite scaffolds

Surgeries provided only one sample of either an otoplasty/normal or microtia remnant and therefore the cells from each patient were unique and separate from the others. Resulting cultured human auricular (otoplasty/normal or microtia) chondrocyte concentrations were adjusted to 1 x 10^8^ cells/ml before seeding the cells from a pipet onto ear-shaped bioresorbable composite polymer scaffolds placed in individual 10 cm Petri dishes. Normal or microtia cells were seeded randomly onto both ethanol-treated and untreated (control) scaffolds. Resulting tissue-engineered constructs of chondrocyte-seeded scaffolds (approximately three constructs per surgery sample; Table 1) in dishes were then removed to an incubator (5% CO_2_, 37°C) for 1 hr to permit cell adherence to scaffolds before further addition of complete culture medium. Cell-seeded scaffolds were incubated for 2–3 days until implantation.

Constructs of chondrocyte-seeded bioresorbable scaffolds (including scaffolds with or without ethanol treatment) were implanted in the subcutaneous dorsal space of athymic mice, one construct per mouse [13]. For pre-anesthesia of the animals, tolbutamide (3 mg/kg) and atropine sulfate (0.04 mg/kg) were subcutaneously injected. This procedure was followed by inhalation with isoflurane (Pittmann and Moores, Mundelein, IL, USA) administered with an anesthesia machine for induction and maintenance of the drug. The dorsal surface was disinfected with povidone-iodine (Purdue Frederick Co., Stamford, CT) and an incision of ~ 2 cm was made. A subcutaneous pocket was created, the construct was inserted, and the wound was closed with 5–0 Vicryl thread (Thermo Fisher Scientific Intl.). Six cell-seeded constructs whose scaffolds were untreated with ethanol and six cell-seeded constructs whose scaffolds were treated with ethanol were initially implanted in nude mice for 10 weeks. During this implantation period, two mice were euthanized because their implanted constructs consisting of scaffolds without ethanol treatment extruded from their skin and caused chronic skin lesions. In this instance with increased loss of mice unable to survive through 10 weeks of implantation, an additional four constructs that were provided ethanol treatment were implanted in mice for 20 weeks. Further, no animals were implanted for 20 weeks for scaffolds without ethanol treatment, and four more constructs that were ethanol-treated were implanted in mice for 10 weeks. In the 20-week implantation group, one animal with an unresolved seroma was euthanized one-week post-surgery. All mice were euthanized by CO_2_ overdose followed by cervical dislocation. In total, construct numbers retrieved for analysis were n = 4 (10 weeks of implantation, ethanol-untreated), n = 10 (10 weeks of implantation, ethanol-treated) and n = 3 (20 weeks of implantation, ethanol-treated).

### Histology of implanted constructs

Constructs were excised from nude mice after 10 or 20 weeks of implantation and bisected immediately after harvest. The upper ear portion of the bisected constructs was placed in RNAlater (Ambion, Thermo Fisher Scientific, Intl.) and frozen at -80°C for nucleic acid preservation and later gene expression analysis, and the remaining lower portion of the same constructs was fixed by immersion in 10% neutral buffered formalin for one week for histological examination. Subsequently, each fixed specimen was dehydrated through a graded ethanol series, paraffin-embedded, and cut with a microtome into 6-μm thick sections from the bisected face in the middle of the ear construct. Safranin-O red (Saf-O) staining was used to determine proteoglycan presence and Verhoeff staining was applied to identify elastic fibers in the thick sections. Saf-O-stained sections were counterstained with fast green to show general morphology.

Two constructs each whose scaffolds had been treated with ethanol and implanted for 10 and 20 weeks were additionally dissected. These four specimens, destined for gene expression analysis, were investigated to examine intact PCL that would normally dissolve during paraffin processing of the constructs. In this instance, a small portion of the four specimens was cut following construct harvest and the samples were immediately frozen in tissue freezing medium (TFM, Thermo Fisher Scientific Intl.). Samples were subsequently frozen-sectioned at 10–12 μm thickness utilizing a Microm HM560 cryostat (Richard Allan Scientific, Kalamazoo, MI), cooled to about -20 to -30°C. Sections were stained with toluidine blue for spatial identification of cells, extracellular matrix and fibrous tissue in relation to the PCL comprising the construct inner core.

### Quantitative histomorphometric evaluation of cartilage matrix regeneration

Cartilage matrix-producing regions in construct sections identified by proteoglycan staining with Saf-O were analyzed using imaging analysis software (Image J; National Institutes of Health). The regenerated cartilage area in a section was determined as the ratio of Saf-O-positive pixels to total scaffold surface area (pixels). The scaffold coverage of regenerated cartilage was also determined as a two-dimensional (2D) linear parameter, evaluated by selecting the periphery of each construct and calculating a ratio of the length of cartilage regeneration (Saf-O-positive staining pixel number) along the perimeter to the total circumference of the scaffold (pixel number). Three sections were measured (Image J) from each construct (n = 5 for 10-week implanted constructs and n = 3 for 20-week implanted constructs) and presented as box plots with mean values and respective standard deviations GraphPad Prism (GraphPad Software Inc., San Diego, CA).

### Quantitative gene expression analysis of construct cells

Expression of genes of interest from the chondrocytes populating the harvested constructs after their implantation and development for 10 or 20 weeks in vivo was determined by RT-qPCR analysis. Previously frozen, bisected upper half portions of auricular chondrocyte/scaffold constructs with newly generated cartilage were prepared by separately grinding the constructs to powders under liquid nitrogen in a Spex 6870 freezer/grinder mill (Spex, Inc., Metuchan, NJ). Total RNA was extracted from the cellular component of the constructs using an E.Z.N.A^™^ Micro Elute Total RNA Kit (Omega Bio-tek Inc., Norcross, GA). The extracted RNA solution (1 μL) and 50 μL of Tris/EDTA buffer (TE buffer) were mixed, and the RNA level was measured using an ultraviolet and visible light spectrophotometer (Eppendorf Bio Photometer; Brinkmann Instruments Inc., Westbury, NY) [7, 37]. Reverse transcription with 20 μl reaction volumes contained 10X reaction buffer (2 μl), RNAse inhibitor (20 units/μl, 0.5 μl), random hexamers (1 μl), oligo-dT primers (1 μl), 10 mM deoxynucleotide triphosphate (dNTPs, 2 μl), Multiscribe reverse transcriptase (1 μl of 50 units/μl,) DEPC-treated water and 1 μg of extracted RNA. Buffer blanks contained the same components without sample RNA for verification of the absence of cDNA contamination. TaqMan^®^ gene expression assay kit and TaqMan^®^ gene expression primer sets were utilized for PCR analysis on an ABI Prism 7500 Sequence Detector Real Time PCR System (Applied Biosystems) following the standard protocol of the manufacturer [7, 37]. Certain genes involved in cartilage matrix formation were analyzed and included type II collagen (*COL2A1*, Hs00264051_m1), elastin (*ELN*, Hs00355783_m1), and SRY (sex determining region Y)-box 5 (*SOX5*, Hs00374709_m1). The inflammatory response gene, interleukin-1α (*IL1A*, Hs00174092_m1), was investigated as were apoptosis genes, caspase-8 (*CASP8*, Hs01018151_m1) and caspase-9 (*CASP9*, Hs00609647_m1). A housekeeping gene, large ribosomal protein P0 (*LRP0*, 4326314E), was used to normalize message from retrieved constructs of the auricular chondrocyte-seeded nanoPGA/PCL composite scaffolds, β2-microglobulin (*B2M*, Hs00187842_m1) was measured to assess possible global differences between sample groups, and 18S rRNA (4352930E) values determined cDNA quantity and quality from experimental samples [7, 37]. Samples of poor RNA quality or quantity were removed from the study. All reagents and primer sets for reverse transcription and PCR were purchased from Applied Biosystems (Thermo Fisher Scientific Intl.).

### Statistical analysis

Significance of differences in cell count analysis was analyzed using a two-tailed student t-test, equal variance, and averages with respective standard deviations were plotted (JMP 14.2.0; SAS Institute Inc., Cary, NC). Quantitative RT-PCR data from ethanol-treated and untreated 10-week constructs were compared using the Wilcoxon test and averages with their respective standard errors of the mean were plotted with GraphPad Prism (GraphPad Software Inc.). Quantitative RT-PCR data from ethanol-treated 10- and 20-week constructs were compared by a two-tailed student t-test with two sample unequal variance (Excel vs. 2102, Microsoft Corp., Redmond, WA) and presented in a table. Quantitative assessment of cartilage matrix histomorphometry was determined utilizing Image J software (National Institutes of Health) and presented as box plots as noted above. Statistical significance for all analyses was considered as p ≤ 0.05.

## Results

**Fig 1:**
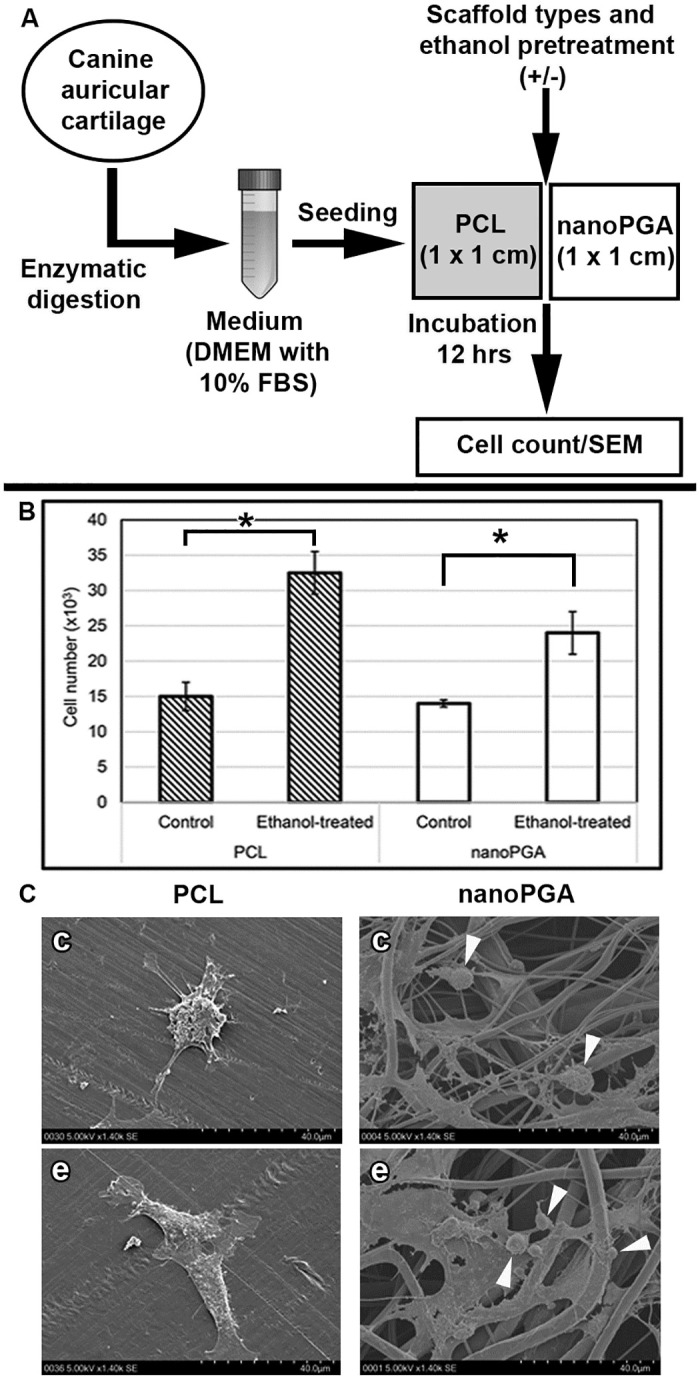
The experimental outline and data generated from the initial study of canine auricular chondrocytes seeded in vitro onto polymer sheets with or without ethanol treatment. (A) A stepwise depiction of the research design from the enzymatic digestion of canine auricular chondrocytes to their seeding onto polymer sheets of either nanoPGA or PCL and final analyses by cell counting and SEM. Six nanoPGA and six PCL sheets received no treatment (control) and an additional six of each of the same sheets were immersed through graded ethanols. (B) Chondrocytes retrieved after trypsinization from polymers were counted following cell seeding on sheets for 12 hr. Statistically significant greater cell counts were found for both ethanol-treated polymer sheets [PCL, n = 4; nanoPGA, n = 4] when compared to their counterpart controls [PCL, n = 4; nanoPGA, n = 4] (p = 0.002 and p = 0.019, respectively). (C) SEM showed flattened cells with prominent filipodia on ethanol-treated (e) PCL sheets (n = 2) and more rounded chondrocytes with limited filipodia visible on control (c) PCL sheets (n = 2). Canine auricular chondrocytes were visible (arrowheads) on nanoPGA sheet fibers with (n = 2) or without (n = 2) ethanol treatment.

The experimental study reported here was begun initially with an investigation in vitro of possible effects of ethanol treatment on the affinity of canine auricular chondrocytes for small sheets of individual hydrophobic PCL and hydrophilic nanoPGA. Based on the outcome of resulting data of such preliminary work, subsequent examination was then conducted of ethanol treatment of composite nanoPGA/PCL scaffolds seeded with human auricular chondrocytes. Fig 1 presents the basic framework of the canine experiment (Fig 1A) and summary information (Fig 1B and 1C) demonstrating the effects of ethanol treatment of the two types of polymer sheets. As a measure of the adhesion of canine auricular chondrocytes to the polymers, the number of cells recovered following their seeding for 12 hr and subsequent trypsinization from polymer sheets was found to increase approximately two-fold with a significant difference (p = 0.002) determined for ethanol-treated PCL sheets compared to untreated PCL sheets (Fig 1B). Adhesion improved approximately 1.7-fold with a significant difference (p = 0.019) obtained for treated compared to untreated nanoPGA sheets (Fig 1B). SEM of the cells seeded onto PCL sheets without ethanol treatment demonstrated that they maintained a spherical shape with few cell processes attached to the sheets, while chondrocytes seeded onto ethanol-treated PCL sheets were highly flattened in shape and extended numerous processes onto the sheets (Fig 1C). Cell attachment was observed on both ethanol-treated and untreated nanoPGA sheets (comprised of small fibers), but distinct morphological changes were not apparent between the two groups (Fig 1C).

**Fig 2:**
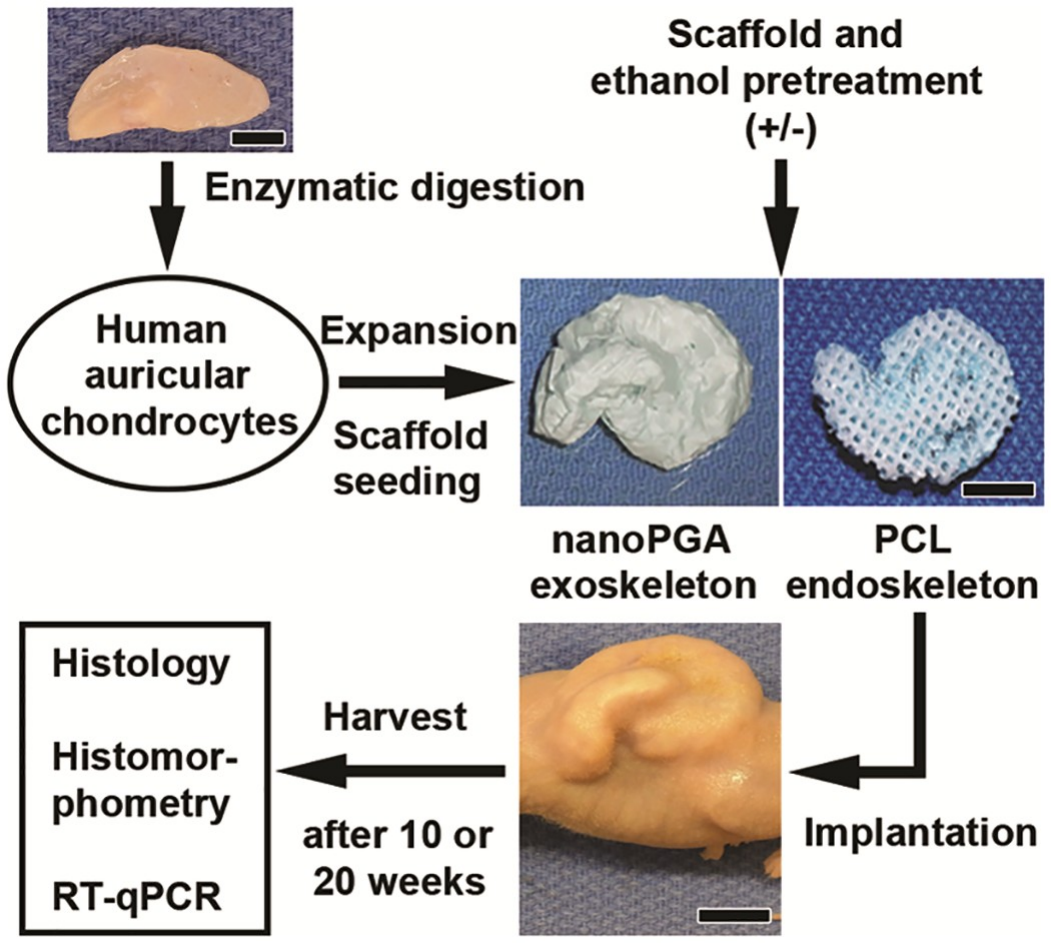
Experimental design for examination of tissue-engineered auricle-shaped constructs of human auricular chondrocytes seeded onto 3D nanoPGA/PCL composite polymeric scaffolds with or without ethanol treatment. The stepwise depiction shows a human surgical biopsy specimen (either normal conchal [otoplasty] or microtia) utilized in the digestion and isolation of auricular chondrocytes. The cells were subsequently expanded in vitro, counted and seeded onto 3D ear-shaped nanoPGA/PCL composite scaffolds consisting of an inner PCL core wrapped in a nanoPGA sheet. Some scaffold composites were treated with graded ethanols before cell seeding and implantation into nude mice for 10 or 20 weeks. On their retrieval, these tissue-engineered constructs were assessed by histology, histomorphometry and RT-qPCR analysis. Scale bars = 1 cm (scaffold, mouse) and 0.5 cm (biopsy tissue).

Fig 2 presents a schematic of the experimental protocol followed for the human surgical auricular tissues examined in this study (Table 1). The outline shows individual steps in procedure from chondrocyte isolation, expansion, and seeding onto nanoPGA/PCL composite scaffolds to retrieval from nude mice after 10 or 20 weeks of implantation in vivo and final data analysis by histological, histomorphometric and gene expression analyses. Auricular chondrocytes were isolated from human otoplasty or microtia patients and seeded onto bioresorbable composite scaffolds consisting of an inner core PCL endoskeleton in the shape of a human auricle and wrapped with a nanoPGA exoskeleton. Seeded composite scaffolds of this type treated with ethanol or identical counterpart scaffolds left untreated were subsequently implanted in the dorsal region of athymic mice. All constructs maintained structural stability and their 3D auricular shape and integrity could be observed beneath the skin of the mouse immediately after and throughout the extent of up to 20 weeks of implantation as illustrated in Fig 2.

**Fig 3:**
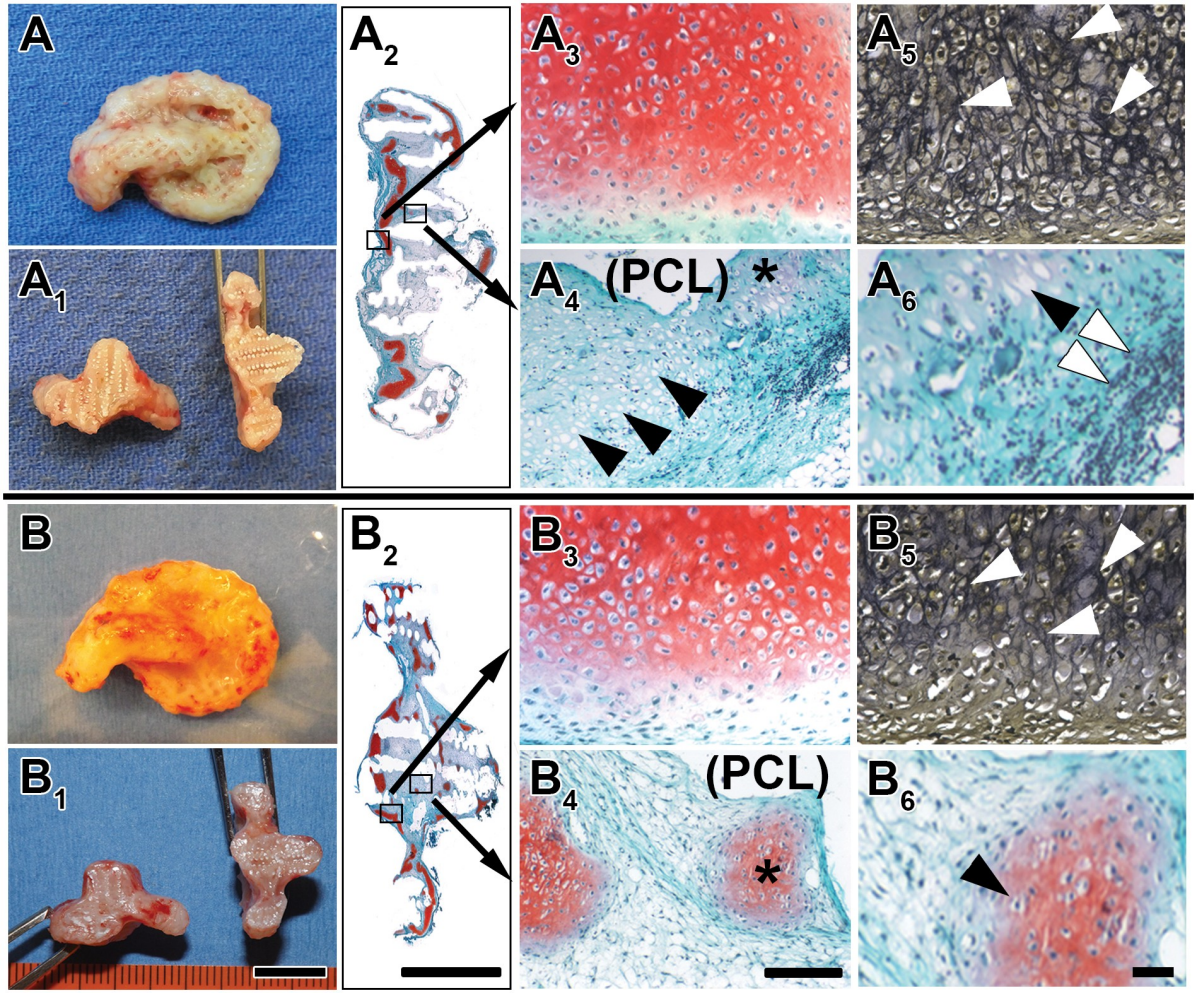
Representative histology of tissue-engineered auricle-shaped constructs comprised of human auricular chondrocytes seeded onto nanoPGA/PCL composite polymeric scaffolds with and without ethanol treatment and harvested following implantation for 10 weeks in nude mice. Gross morphology and histology of representative intact and bisected auricle-shaped constructs with their scaffolds either untreated (A-A6) or ethanol-treated (B-B6) prior to cell seeding and construct implantation in nude mice for 10 weeks. Glossy, white regenerated cartilage was more evident in ethanol-treated constructs (B and B1) when compared with their counterpart control constructs whose scaffolds were not subject to ethanol immersion (A and A1). Sections of typical constructs showed dissolution of core PCL as a result of specimen processing, leading to a checkered pattern of empty spaces (A2 and B2). Some aspects of the interior structure of these specimens remained partially intact and Saf-O and Verhoeff staining revealed the presence of proteoglycans and elastic fibers, respectively (A2 and B2). Saf-O staining was most evident at the periphery of sections and more abundant in constructs whose scaffolds were ethanol-treated (B2) compared to untreated (A2). Selected areas marked by small boxes (A2 and B2) were enlarged to show histological features more clearly. Proteoglycans (A3) and elastic fibers (A5, white arrowheads) were apparent in constructs comprised of untreated scaffolds and such specimens also consisted of fibrous tissue counterstained by fast green (A4, blue-green) near spaces created by PCL dissolution (A4, (PCL)), numerous empty chondrocyte lacunae (A4, black arrowheads), and small pockets of pale Saf-O staining (A4, asterisk). On greater enlargement (A6), empty lacunae (black arrowhead) and cellular infiltration (white arrowheads) were observed in sections. Constructs comprised of polymer scaffolds treated with ethanol maintained qualitatively more prominent proteoglycans stained with Saf-O (B3), many lacunae enclosing chondrocytes (B3) and distinctive elastic fibers revealed by Verhoeff staining (B5). Additionally, such specimens were found with fibrous tissue (B4, blue-green) neighboring PCL-devoid spaces (B4, (PCL)), areas of qualitatively greater Saf-O staining (B4, asterisk) compared to counterpart sections from constructs without ethanol exposure (A4), and, on enlargement, several chondrocytes within their lacunae (B6, black arrowhead). Cell infiltrates were typically absent from constructs whose scaffolds were ethanol-treated (B4 and B6). Scale bars = 1 cm (B1 [A, A1, and B at the same enlargement]), 1 cm (B2 [A2]), 200 μm (B4 [A3-5, B3, and B5]), 50 μm (B6 [A6]).

On retrieval from mice after 10 weeks of implantation, auricular constructs whose scaffolds were not treated with ethanol were characterized by thin connective tissue with irregular cartilage regeneration, identified grossly by its glossy appearance, localized about the auricle template (Fig 3A). Dissected transversely, such constructs were found with little developed cartilage beneath the specimen surface (Fig 3A1). Following Saf-O and Verhoeff staining of dissected sections, these constructs were marked with proteoglycans (Fig 3A2 and 3A3) and elastic fibers (Fig 3A5), respectively, confined principally to peripheral regions of the constructs. Interior regions of the constructs were generally devoid of Saf-O and Verhoeff staining and PCL was largely dissolved from the constructs during their processing, leaving empty spaces (Fig 3A2). At some interior sites, tissue was present near such spaces and it was typified by the presence of fibrous material, empty chondrocyte lacunae, weak staining for proteoglycans, and evidence of large-scale infiltration by inflammatory cells (Fig 3A4 and 3A6).

Over the same 10-week implantation period, auricular constructs comprised of scaffolds treated with ethanol were heavily covered with glossy cartilage (Fig 3B) that extended well within the auricle template as observed in transverse profiles (Fig 3B1). An additional distinction from their counterpart control constructs (scaffolds that were not ethanol-treated), sections of constructs whose scaffolds were treated with ethanol maintained greater areas of Saf-O and Verhoeff staining (Fig 3B2), which produced qualitatively richer levels of proteoglycans (Fig 3B3 vs Fig 3A3) and elastic fibers (Fig 3B5 vs Fig 3A5). The interior regions of constructs with ethanol-treated scaffolds maintained numerous chondrocytes residing in their lacunae near PCL-devoid areas and many pockets of strong proteoglycan staining, unlike those regions from constructs with scaffolds left untreated with ethanol (Fig 3B4 and 3B6 vs Fig 3A4 and 3A6, respectively). Constructs whose scaffolds were ethanol-treated also showed little evidence of inflammation by infiltrating cells (Fig 3B4 and 3B6).

**Fig 4:**
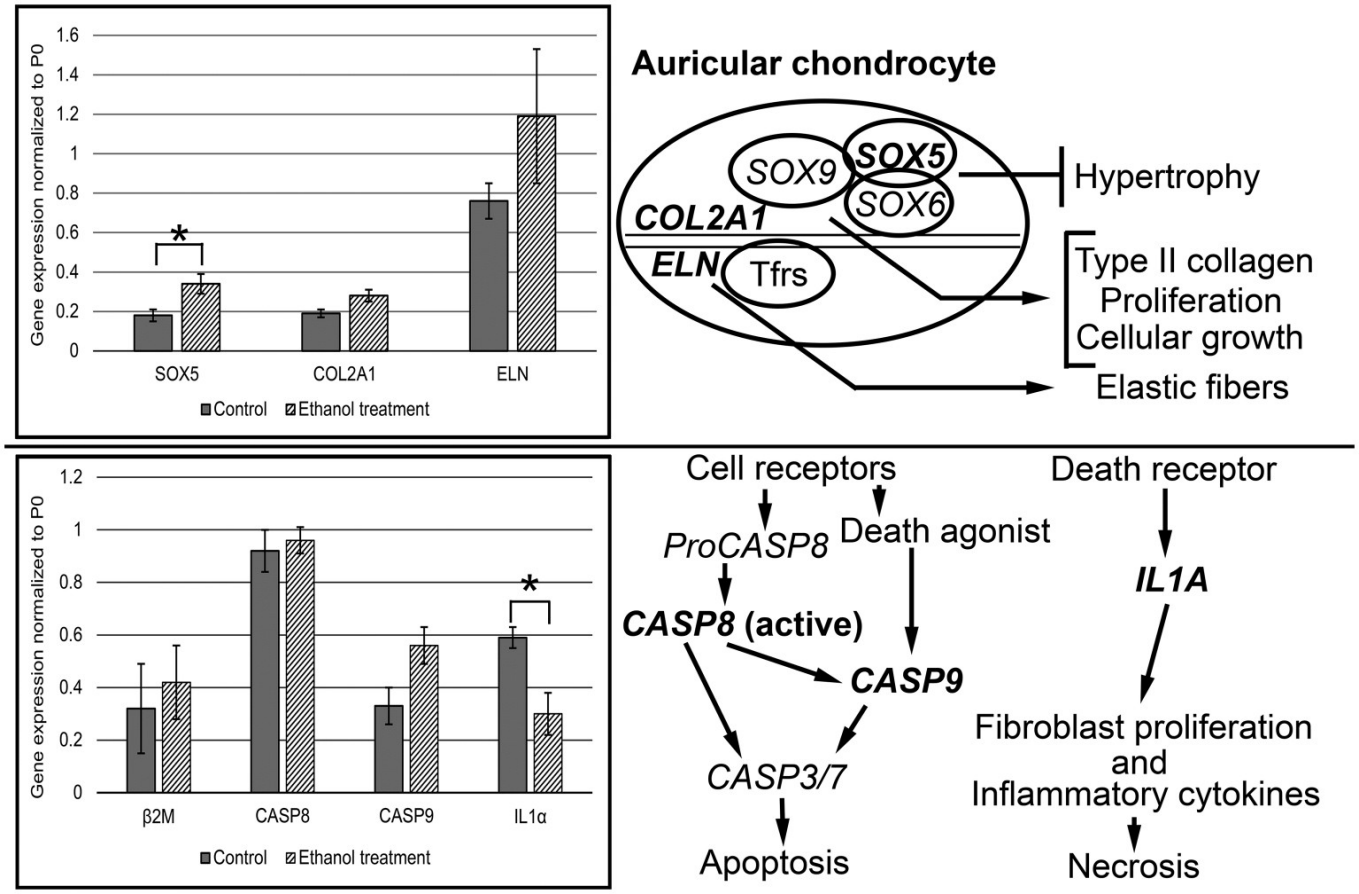
Plots of gene expression values for *SOX5*, *COL2A1*, *ELN*, *B2M*, *CASP8*, *CASP9*, and *IL1A* normalized to large ribosomal fraction, *P0*, obtained from RT-qPCR analysis of constructs comprised of human auricular chondrocytes seeded onto ethanol-treated or untreated nanoPGA/PCL composite scaffolds and schematics of gene function and apoptosis/necrosis pathways. Following specimen implantation in nude mice for 10 weeks, comparisons were made between tissue-engineered human auricular constructs having their scaffolds either treated with ethanol or left untreated (controls). For treated (specimen number, n = 6) compared to control (n = 4) specimens, quantitative chondrocyte expression levels were found to be significantly increased (*p = 0.040) for *SOX5* and equivalent (not statistically significantly different) for *COL2A1* (p = 0.080) and *ELN* (p = 0.331) (upper left panel). There were no significant differences between ethanol-treated and untreated constructs in gene expression of *B2M* (p = 0.187), *CASP8* (p = 0.737) or *CASP9* (p = 0.070) while *IL1A* was significantly decreased (*p = 0.040) (lower left panel). Functionally, viable auricular chondrocytes express genes (boldface type) with appropriate transcription factors (Tfrs, *SOX9*) that lead to the translation of proteins necessary for formation of a cartilaginous extracellular matrix (upper panel right). Cells and tissues normally follow apoptotic and necrotic pathways through different gene pathways (lower right panel). Certain of these genes (boldface type) analyzed in this study are shown in such pathways. These genes represented the major and definitive pathway genes of principal interest in this report, and they precluded examination of additional genes (such as *SOX6*, *SOX9*, *CASP3*, *CASP7*) of slightly less importance in the work or involved in secondary pathways. Error bars represent standard error of the mean for normalized gene expression values. *SOX6* or *SOX9* = SRY (sex determining region Y)-box 6 or 9.

Expression of cartilage-related (*COL2A1*, *ELN*, and *SOX5*), apoptosis-related (*CASP8*, *CASP9*), inflammation-related (*IL1A*) and reference (*P0*, *B2M*) genes (Fig 4) in regenerated auricular tissue was investigated using RT-qPCR analysis. Ethanol treatment of scaffolds comprising tissue-engineered constructs led to increased expression of all cartilage-related genes (Fig 4, upper left plot) compared to untreated scaffolds of counterpart constructs (controls). In particular, ethanol treatment of scaffolds induced significantly greater (p = 0.040) *SOX5* gene expression when compared to controls after 10 weeks of construct implantation. No statistically significant changes were noted in any other genes examined in this study (Fig 4, upper and lower left plots) except for that of *IL1A*. In this case, *IL1A* gene expression was significantly decreased (p = 0.040) in ethanol-treated constructs when compared to untreated controls after 10 weeks of implantation. Schematics of basic functions of the genes analyzed in this work are presented for auricular chondrocytes leading to cartilage extracellular matrix formation (Fig 4, upper right panel) as well as pathways for cellular apoptosis and tissue necrosis (Fig 4, lower right panel) [39–41].

**Fig 5:**
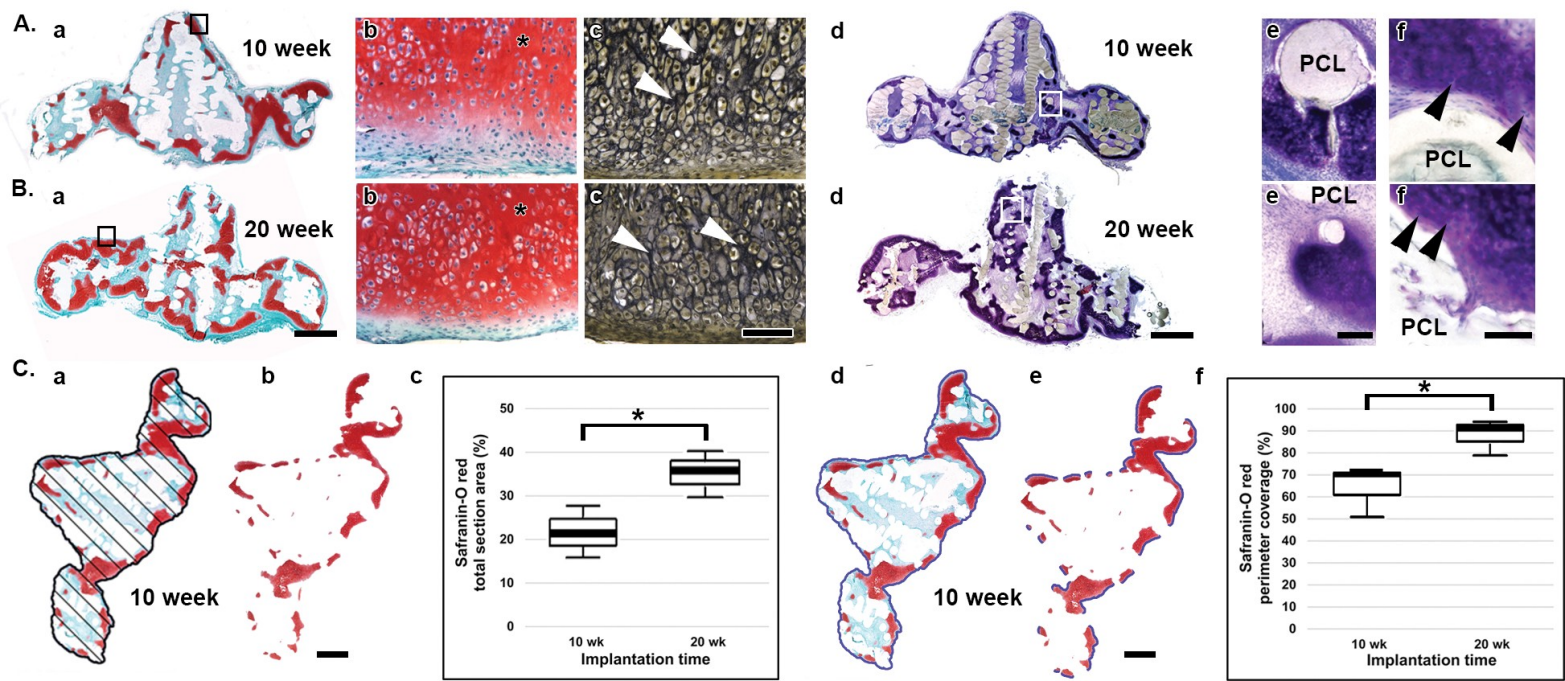
Representative histology and histomorphometry of human auricular cartilage regenerated on nanoPGA/PCL composite constructs consisting of ethanol-treated scaffolds and implanted for 10 and 20 weeks in nude mice. A, B and C show representative complete cross sections of constructs that were fixed, processed in paraffin and stained with Saf-O for the presence of cartilage proteoglycans. Other such sections were Verhoeff-stained for the presence of elastic fibers. Thick sections of constructs implanted for 10- (Aa) or 20- (Ba) weeks showed Saf-O staining along the periphery of the specimens with more staining evident after the longer implantation times. Enlargement of typical construct areas (Aa or Ba, black-outlined boxes) demonstrated abundant proteoglycans (asterisks) and numerous chondrocytes in lacuna (Ab and Bb) as well as elastic fibers in other corresponding construct sections (Ac and Bc, white arrows). Completely unfixed, frozen constructs that were sectioned and stained with toluidine blue revealed prominent metachromasia, indicative of cartilage, in interior regions of specimens (Ad and Bd). Cartilage was more apparent in constructs implanted for 20 weeks (Bd) than 10 weeks (Ad) and the histology correlated well with that of sections stained with Saf-O (Aa and Ba). On enlargement of certain areas (Ad or Bd, white-outlined boxes), intact PCL cores were bounded by metachromatic cartilage (Ae and Be) and presumptive chondrocytes (Af and Bf, black arrows) without evidence of inflammatory elements. Based on Saf-O presence, histomorphometric measurements were made and plotted to show percent total section area (Ca-c) and percent perimeter coverage (Cd-f). A representative Saf-O-stained section for a construct implanted for 10 weeks details two imaging steps in a calculation for Saf-O total area (Ca-b) and Saf-O perimeter coverage percent (Cd-e). A set of evenly spaced straight lines was used in total pixel area measures (Ca) and then non-staining pixel areas were subtracted to leave only Saf-O-stained pixel domains (Cb). In a related manner, a line (Cd, blue) circumscribing a scaffold was used to measure total scaffold circumference in pixels. Saf-O-stained aspects only along the same line were identified (Ce) and their total length in pixels was compared to the total circumference. These measures provide assessment of cartilage regenerated during construct implantation. Resulting box plots show significant increases (*, p = 0.044 and p = 0.034, respectively) from 10 to 20 weeks of implantation of Saf-O total area percent (Cc) and perimeter coverage percent (Cf). Scale bars = 2 mm (Ba [Aa at the same enlargement]), 100 μm (Bc [Ab-c, Bb]), 2 mm (Bd [Ad]), 100 μm (Be [Ae]), 100 μm (Bf [Af]), 2 mm (Cb [Ca]) and 2 mm (Ce [Cd]).

Fig 5 presents several results comparing constructs retrieved from 10 and 20 weeks of implantation in nude mice in order to study longer term effects of ethanol treatment on scaffolds. Control constructs whose scaffolds were not treated with ethanol supported cells that were characterized by a loss of the chondrocyte phenotype and also led to scaffold extrusion through the skin of the host mice, seromas and premature death of mice after just 10 weeks of implantation. The consequence of these changes resulted in unsustainable viable cartilage over longer implantation times and discontinuation of constructs comprised of ethanol-untreated scaffolds to 20-week implantation time points.

Saf-O-positive regions observed from cross sections after 10 weeks in vivo (Fig 5Aa) were confined predominantly along the surface of ethanol-treated scaffolds compared to surface and interior Saf-O staining after 20 weeks of implantation (Fig 5Ba). Cartilage regeneration after 10- or 20-week end points was characterized by rich proteoglycan presence, numerous chondrocytes in lacuna and prominent elastic fibers (Fig 5Ab, 5Ac, 5Bb and 5Bc). Unfixed, toluidine blue-stained frozen sections of constructs identified areas of cartilage regeneration around intact PCL cores without indication of an inflammatory response (Fig 5Ad–5Af and 5Bd–5Bf). Histomorphometry of the area percent of regenerated cartilage (Fig 5Cc) and perimeter coverage percent (Fig 5Cf) determined by Saf-O staining (Fig 5Ca, 5Cb, 5Cd and 5Ce, respectively) showed that cartilage-regenerated area percent was significantly increased (p = 0.044) approximately 15% from 10 to 20 weeks of implantation (Fig 5Cc). The perimeter coverage percent was also significantly increased (p = 0.034) approximately 20% over the same time period. Further analyses of 10- and 20-week implanted specimens with chondrocytes seeded onto ethanol-treated scaffolds demonstrated maintenance or an increase in expression levels for certain extracellular matrix genes (Table 2). There was a significant increase found for expression of *COL2A1* from 10 to 20 weeks (p = 0.017, Table 2). Additionally, the apoptotic-related genes, *CASP8* or *CASP9*, did not change statistically significantly over the same time frame while the inflammation-related gene, *IL1A*, significantly decreased in its expression levels (Table 2).

**Table 2:** Average gene expression values normalized to P0 for human auricular chondrocytes seeded onto ethanol-treated scaffolds and retrieved from tissue-engineered constructs after 10- or 20-weeks of implantation in nude mice.

Gene	10-week	20-week	p value
	Average ± SEM	Average ± SEM	
	n = 5	n = 3	
β2m	0.42 ± 0.14	0.47 ± 0.06	0.511
SOX5	0.34 ± 0.05	0.49 ± 0.08	0.062
Type II collagen	0.28 ± 0.03	0.52 ± 0.06	0.017
Elastin	1.19 ± 0.34	1.13 ± 0.17	0.692
Caspase-8	0.96 ± 0.05	0.96 ± 0.10	0.661
Caspase-9	0.56 ± 0.07	0.62 ± 0.11	0.201
Interleukin-1α	0.30 ± 0.08	0.18 ± 0.06	0.076

p values are t-test comparisons between 10- and 20-week data. Gene expression levels at 10-week implantation times are presented graphically in Fig 4 compared to control constructs. Key: SEM = standard error of mean.

## Discussion

This study sought to advance the often difficult surgical procedure of auricle reconstruction. The work began with a tissue-engineering approach utilizing a novel composite polymeric scaffold of nanoPGA/PCL on which to seed human auricular chondrocytes obtained following otoplasty of normal (conchal) or microtic tissue. Earlier investigations from this and other laboratories have established the experimental basis for such an avenue with auricular cells grown in hydrogels or on a variety of polymers designed as sheets, discs or 3D ear-shaped scaffolds [4–6]. The present examination follows more recent reports that human conchal and microtia chondrocytes are equivalent and may each be utilized experimentally to regenerate tissue-engineered auricular cartilage [7–9].

Equally important to the several factors mentioned previously in this report that influence cell growth and development in tissue engineering, the characteristics of the microenvironment presented to the cells are essential for their viability and maintenance. Simulating in vitro as closely as possible the natural state of a cell and its microenvironment in vivo is a critical determinant for engineered tissue viability and regeneration over long time periods. As cellular substrates that are intended to provide an architectural framework with adequate biomechanical properties, conventional polymers like PGA and PCL, commonly utilized in the tissue engineering field, may be acceptable for certain applications, but each has significant limitations. For example, PGA, and less so PCL, leads to an increase in acidity on its degradation during hydrolysis and bioresorption [29, 30]. Release of glycolic acid in these processes has been reported in cartilage to induce a high localized pH in the chondrocyte microenvironment that induces inflammation and disrupts local tissue and its regeneration [22, 30]. PCL, however, is hydrophobic compared to the more biocompatible nature of hydrophilic PGA [29, 30].

With regard to the unique nanoPGA/PCL scaffolds utilized in the present study, PGA nanofibers replacing conventional PGA reduce the inflammatory response as a result of a shortened resorption time in the body compared to standard PGA, and nanoPGA may promote chondrocyte adhesion and proliferation because its composite nanofibers resemble the size and structure of the natural extracellular matrix of cartilage [28, 42]. The use of ethanol as a treatment for both nanoPGA and PCL prior to chondrocyte seeding of individual sheets of these polymers as well as of 3D-fabricated composite auricular-shaped polymer constructs was examined as a possible means of improving the hydrophilicity and biocompatibility principally of PCL but also of nanoPGA.

Initial experiments with ethanol treatment of separate polymer sheets were conducted in vitro with canine auricular chondrocytes because of their availability compared to human auricular cells. In the absence of information related to the adherence of these cells to the sheets, chondrocytes were purposefully over-seeded at high concentration so that maximal cell numbers attached to the polymers. Such over-seeding accounted for differences between the number of cells applied initially to the scaffolds (10^5^) and those ultimately counted on the sheets (~ 10^4^).

The preliminary results with such canine cells, revealing qualitatively more numerous cell processes and statistically significantly increased cell adhesion for chondrocytes seeded onto ethanol-treated nanoPGA and PCL sheets compared to those cells incubated on ethanol-untreated sheets, supported the concept that ethanol promoted the wettability of each polymer and enhanced its hydrophilic nature or properties. These data justified further investigation utilizing human auricular chondrocytes.

Studies with human cells yielded results relevant to ethanol treatment of polymer scaffolds that were consistent with those obtained with canine chondrocytes. In this instance, on retrieval after 10 weeks of implantation in nude mice, the tissue-engineered 3D composite nanoPGA/PCL constructs comprised of scaffolds treated with ethanol showed several differences on comparison with the same cells seeded onto constructs consisting of ethanol-untreated scaffolds. The former constructs were found with improved cartilage regeneration marked by increased Saf-O staining for proteoglycans in peripheral and interior core construct regions, abundant numbers of chondrocytes residing within their cell lacunae, absence of cellular infiltrates and inflammation, and statistically significantly greater gene expression levels of *SOX5* and lower levels of *IL1A*. *SOX5* is a transcription factor reflecting maintenance of a chondrocyte phenotype and inhibiting hypertrophy [40], and *IL1A* is indicative of a cell or tissue inflammatory response and limits proteoglycan synthesis [41, 43]. Constructs designed with polymer scaffolds that were left without ethanol treatment maintained cartilage that was poorly staining for proteoglycans, had numerous empty chondrocyte lacunae, and showed evidence of cellular infiltration. Although this study did not determine hydrophilicity directly, the summary of these results leads to the possibility that ethanol treatment enhanced hydrophilicity of the scaffolds comprising nanoPGA/PCL composite constructs. This consideration would underscore the enhancement of cell attachment to these constructs and improve the microenvironment for chondrocyte-mediated cartilage growth and development during tissue engineering of a 3D auricle over 10 weeks of implantation in nude mice.

Treating the nanoPGA/PCL construct scaffolds with an ethanol step prior to human auricular cell seeding yielded subsequent progression of regenerated cartilage tissue over 20-week timeframes following implantation in vivo. In this case, compared to 10-week harvested constructs, specimens retrieved after 20 weeks were found with statistically significantly greater cartilage regeneration about the perimeter and total area of tissue sections stained by Saf-O and proteoglycans. The presence in frozen sections from both 10- and 20-week constructs (and more so in the older constructs) of prominent toluidine blue metachromasia about intact PCL polymer may be indicative of an ethanol-treated scaffold material that results in limited acidity and damage to chondrocytes and cartilage and the normal deposition or replacement of tissue by healthy regenerated cartilage. Such a supposition would be consistent with the observed maintenance of expression levels at 20- compared to 10-week constructs for genes responsible for the cartilage phenotype and secretion of an auricular cartilage extracellular matrix, including increased expression of both *SOX5* and *COL2A1*, the latter being statistically significant. Additionally, over the same time period, there were no changes in apoptotic genes and a decrease in inflammatory gene expression. These findings again lend themselves to the conclusion that ethanol treatment of polymer scaffolds improves the microenvironment of chondrocytes and cartilage during tissue-engineering events leading to auricular cartilage regeneration over time.

PGA and PCL degradation on hydrolysis and bioresorption leads to an increase in acidity in the microenvironment of cell-seeded constructs fabricated by tissue engineering methods [22, 29, 30]. The higher acid content may in turn result in inflammation and cell death [34, 35]. In this context, the current study concerned itself in part with an analysis of the principal inflammatory gene, *IL1A*, as well as major apoptosis genes, *CASP8* and *CASP9*, to gain possible insight into the role of these molecules in chondrocyte viability in the auricle constructs described here. Necrosis is presently recognized as a basic pathway that may result in cell death; apoptosis is programmed cell death [39–41]. The necrosis pathway to premature cell death involves deleterious external stimuli, inflammation and factors related to the interleukin family of genes while the apoptotic pathway of programmed cell death includes *CASP8* and *CASP9* as certain early, important gene pathway participants. On comparing normalized expression levels from chondrocyte-seeded constructs consisting of ethanol-treated or untreated polymer scaffolds after 10 weeks of implantation in nude mice, the observations that *IL1A* expression per cell is statistically significantly diminished and *SOX5* is statistically significantly increased following ethanol exposure suggest that any disruptive or damaging effects exerted by nanoPGA/PCL on the cells are greatly reduced on ethanol treatment. Further, expression levels of *CASP8* and *CASP9* are essentially unchanged for constructs that are comprised of ethanol-treated or untreated polymer scaffolds. Thus, it may be reasonable to conclude that apoptosis is not an effective cause of cell death compared to inflammation and necrosis, which can be appreciably controlled and limited by ethanol treatment. Further, with ethanol treatment of scaffolds, constructs after 20 weeks of implantation show gene expression statistically significantly increased for *COL2A1*, equivalent for *CASP8* and *CASP9* and decreased for *IL1A* when compared to 10-week ethanol-treated scaffolds. These results support continued active cartilage regeneration over time in constructs comprised of ethanol-treated scaffolds.

The preference and attachment by cells to hydrophilic surfaces having accessible hydrogen groups has been recognized for many years and the bioresorbable or biodegradable polymers commonly utilized in tissue engineering have centered on this knowledge [44]. The high degree of hydrophobicity with limited available hydrogens characterizing most slowly resorbing polymers inhibits cell adherence, an effect that requires modification of their surface with a protein coating of fibronectin or collagen, for example, plasma polymerization or use of porogens to promote hydrophilicity and cell attachment [45–47]. Hydrophilization or prewetting polymers with ethanol has been used routinely to increase polymer hydrophilicity. While the molecular mechanism of action in this context is not entirely clear, ethanol is thought to remove trapped air as it infiltrates spaces in the polymer surface to create a favorable liquid layer with the surface [48]. Ethanol, an amphiphilic molecule, may also act in a manner similar to that of the natural hydrolysis of a polymer, exposing more hydrogen sites available for bonding [29, 32] or possibly altering polymer surface topography [45]. Ethanol has as well been reported to change the permeability and ultrastructure of certain polymer membranes to increase their hydrophilicity [32]. The microenvironment thus becomes more supportive for viable cell attachment, subsequent expression of appropriate genes and secretion of extracellular matrix and other molecules with the water layer decreasing deleterious effects of acidic byproducts generated by polymer degradation. Ethanol treatment of scaffolds comprising nanoPGA/PCL auricular constructs appears to be a straightforward, inexpensive methodology for enhancing cartilage regeneration for tissue engineering purposes and could serve as a model approach for other tissue applications that could lead to improved clinical outcomes. Ethanol washing can be an integral part of the production and manufacture of polymer scaffolds and such ethanol-treated nanoPGA/PCL composites are now being utilized in further tissue-engineering experiments in this laboratory.

## References

[1] Vacanti JP, Morse MA, Saltzman WM, Domb AJ, Perez-Atayde A, Langer R. Selective cell transplantation using bioabsorbable artificial polymers as matrices. J Pediatr Surg. 1988;23: 3–9. doi: 10.1016/s0022-3468(88)80529-3 2895175

[2] Langer R, Vacanti JP. Tissue engineering. Science. 1993;260: 920–6. doi: 10.1126/science.8493529 8493529

[3] Cao Y, Vacanti JP, Paige KT, Upton J, Vacanti CA. Transplantation of chondrocytes utilizing a polymer-cell construct to produce tissue-engineered cartilage in the shape of a human ear. Plast Reconstr Surg. 1997;100: 297–302. doi: 10.1097/00006534-199708000-00001 9252594

[4] Zhou L, Pomerantseva I, Bassett EK, Bowley CM, Zhao X, Bichara DA, et al. Engineering ear constructs with a composite scaffold to maintain dimensions. Tissue Eng Part A. 2011;17: 1573–1581. doi: 10.1089/ten.TEA.2010.0627 21284558

[5] Cohen BP, Hooper RC, Puetzer JL, Nordberg R, Asanbe O, Hernandez KA, et al. Long-term morphological and microarchitectural stability of tissue-engineered, patient-specific auricles in vivo. Tissue Eng Part A. 2016;22: 461–468. doi: 10.1089/ten.TEA.2015.0323 26847742 PMC4800266

[6] Zopf DA, Flanagan CL, Mitsak AG, Brennan JR, Hollister SJ. Pore architecture effects on chondrogenic potential of patient-specific 3-dimensionally printed porous tissue bioscaffolds for auricular tissue engineering. Int J Pediatr Otorhinolaryngol. 2018;114: 170–174. doi: 10.1016/j.ijporl.2018.07.033 30262359 PMC6196359

[7] Nakao H, Jacquet RD, Shasti M, Isogai N, Murthy AS, Landis WJ. Long-term comparison between human normal conchal and microtia chondrocytes regenerated by tissue engineering on nanofiber polyglycolic acid scaffolds. Plast Reconstr Surg. 2017;139: 911e–921e. doi: 10.1097/PRS.0000000000003201 28350666

[8] Childs RD, Nakao H, Isogai N, Murthy A, Landis WJ. An analytical study of neocartilage from microtia and otoplasty surgical remnants: A possible application for BMP7 in microtia development and regeneration. PLoS One. 2020;15: e0234650. doi: 10.1371/journal.pone.0234650 32555733 PMC7299323

[9] Ishak MF, See GB, Hui CK, Abdullah Ab, Saim Lb, Saim Ab, et al. The formation of human auricular cartilage from microtic tissue: An in vivo study. Int J Pediatr Otorhinolaryngol. 2015;79: 1634–1639. doi: 10.1016/j.ijporl.2015.06.034 26250439

[10] Zhou G, Jiang H, Yin Z, Liu Y, Zhang Q, Zhang C, et al. In vitro regeneration of patient-specific earshaped cartilage and its first clinical application for auricular reconstruction. EBioMedicine. 2018; 28: 287–302. doi: 10.1016/j.ebiom.2018.01.011 29396297 PMC5835555

[11] Liao HT, Zheng R, Liu W, Zhang WJ, Cao Y, Zhou G. Prefabricated, ear-shaped cartilage tissue engineering by scaffold-free porcine chondrocyte membrane. Plast Reconstr Surg. 2015;135: 313e–321e. doi: 10.1097/PRS.0000000000001105 25626816

[12] Xu JW, Zaporojan V, Peretti GM, Roses RE, Morse KB, Roy AK, et al. Injectable tissue-engineered cartilage with different chondrocyte sources. Plast Reconstr Surg. 2004;113: 1361–1371. doi: 10.1097/01.prs.0000111594.52661.29 15060348

[13] Kusuhara H, Isogai N, Enjo M, Otani H, Ikada Y, Jacquet R, et al. Tissue engineering a model for the human ear: Assessment of size, shape, morphology, and gene expression following seeding of different chondrocytes. Wound Repair Regen. 2009;17: 136–146. doi: 10.1111/j.1524-475X.2008.00451.x 19152661

[14] Cohen BP, Bernstein JL, Morrison KA, Spector JA, Bonassar LJ. Tissue engineering the human auricle by auricular chondrocyte-mesenchymal stem cell co-implantation. PLoS One. 2018;13: e0202356. doi: 10.1371/journal.pone.0202356 30356228 PMC6200177

[15] Puelacher WC, Kim SW, Vacanti JP, Schloo B, Mooney D, Vacanti CA. Tissue-engineered growth of cartilage: The effect of varying the concentration of chondrocytes seeded onto synthetic polymer matrices. Int J Oral Maxillofac Surg. 1994;23: 49–53. doi: 10.1016/s0901-5027(05)80328-5 8163862

[16] Kim J, McKee JA, Fontenot JJ, Jung JP. Engineering tissue fabrication with machine intelligence: Generating a blueprint for regeneration. Front Bioeng Biotechnol. 2019;7: 443. doi: 10.3389/fbioe.2019.00443 31998708 PMC6967031

[17] Odabas S, Feichtinger GA, Korkusuz P, Inci I, Bilgic E, Yar AS, et al. Auricular cartilage repair using cryogel scaffolds loaded with BMP-7-expressing primary chondrocytes. J Tissue Eng Regen Med. 2013; 7: 831–840. doi: 10.1002/term.1634 23281155

[18] Isogai N, Morotomi T, Hayakawa S, Munakata H, Tabata Y, Ikada Y, et al. Combined chondrocyte-copolymer implantation with slow release of basic fibroblast growth factor for tissue engineering an auricular cartilage construct. J Biomed Mater Res A. 2005;74: 408–418. doi: 10.1002/jbm.a.30343 15973729

[19] Pearson RG, Bhandari R, Quirk RA, Shakesheff KM. Recent advances in tissue engineering. J Long Term Eff Med Implants. 2017;27(2–4):199–231. doi: 10.1615/JLongTermEffMedImplants.v27.i2-4.70 29773040

[20] Peretti GM, Randolph MA, Zaporojan V, Bonassar LJ, Xu JW, Fellers JC, et al. A biomechanical analysis of an engineered cell-scaffold implant for cartilage repair. Ann Plast Surg. 2001;46: 533–537. doi: 10.1097/00000637-200105000-00013 11352428

[21] Roy R, Kohles SS, Zaporojan V, Peretti GM, Randolph MA, Xu J, et al. Analysis of bending behavior of native and engineered auricular and costal cartilage. J Biomed Mater Res A. 2004;68: 597–602. doi: 10.1002/jbm.a.10068 14986315

[22] Sittinger M, Reitzel D, Dauner M, Hierlemann H, Hammer C, Kastenbauer E, et al. Resorbable polyesters in cartilage engineering: affinity and biocompatibility of polymer fiber structures to chondrocytes. J Biomed Mater Res. 1996;33: 57–63. 8736023 10.1002/(SICI)1097-4636(199622)33:2<57::AID-JBM1>3.0.CO;2-K

[23] Bhardwaj G, Webster TJ. Enhanced chondrocyte culture and growth on biologically inspired nanofibrous cell culture dishes. Int J Nanomedicine. 2016;11: 479–483. doi: 10.2147/IJN.S94000 26917958 PMC4751894

[24] Morotomi T, Wada M, Uehara M, Enjo M, Isogai N. Effect of local environment, fibrin, and basic fibroblast growth factor incorporation on a canine autologous model of bioengineered cartilage tissue. Cells Tissues Organs. 2012;196: 398–410. doi: 10.1159/000336029 22677647

[25] Shafiee A, Atala A. Tissue engineering: Toward a new era of medicine. Annu Rev Med. 2017;68: 29–40. doi: 10.1146/annurev-med-102715-092331 27732788

[26] Itani Y, Asamura S, Matsui M, Tabata Y, Isogai N. Evaluation of nanofiber-based polyglycolic acid scaffolds for improved chondrocyte retention and in vivo bioengineered cartilage regeneration. Plast Reconstr Surg. 2014;133: 805e–813e. doi: 10.1097/PRS.0000000000000176 24867739

[27] Tuan RS, Chen AF, Klatt BA. Cartilage regeneration. J Am Acad Orthop Surg. 2013;21: 303–311. doi: 10.5435/JAAOS-21-05-303 23637149 PMC4886741

[28] Haidar MK, Eroglu H. Nanofibers: new insights for drug delivery and tissue engineering. Curr Top Med Chem. 2017;17: 1564–1579. doi: 10.2174/1568026616666161222102641 28017155

[29] Woodruff MA, Hutmacher DW. The return of a forgotten polymer-polycaprolactone in the 21st century. Prog Polym Sci. 2010;35: 1217–1256. doi: 10.1016/j.progpolymsci.2010.04.002

[30] Gorth D, Webster TJ. Matrices for tissue engineering and regenerative medicine. In: Lysaght M, Webster TJ, editors. Biomaterials for artificial organs. Woodhead Publishing Series: Biomaterials; 2011. pp. 270–286.

[31] Enjo M, Terada S, Uehara M, Itani Y, Isogai N. Usefulness of polyglycolic acid-polypropylene composite scaffolds for three-dimensional cartilage regeneration in a large-animal autograft model. Plast Reconstr Surg. 2013;131: 335e–342e. doi: 10.1097/PRS.0b013e31827c6dd8 23446582

[32] Bridge MJ, Broadhead KW, Hlady V, Tresco PA. Ethanol treatment alters the ultrastructure and permeability of PAN-PVC hollow fiber cell encapsulation membranes. J Membrane Science. 2002;195: 51–64. doi: 10.1016/S0376-7388(01)00523-3

[33] Ainslie KM, Tao SL, Popat KC, Daniels H, Hardev V, Grimes CA, et al. In vitro inflammatory response of nanostructured titania, silicon oxide, and polycaprolactone. J Biomed Mater Res A. 2009;91: 647–655. doi: 10.1002/jbm.a.32262 18988278

[34] Valente CA, Chagastelles PC, Nicoletti NF, Garcez GR, Sgarioni B, Herrmann F, et al. Design and optimization of biocompatible polycaprolactone/poly (l-lactic-co-glycolic acid) scaffolds with and without microgrooves for tissue engineering applications. J Biomed Mater Res A. 2018;106: 1522–1534. doi: 10.1002/jbm.a.36355 29388321

[35] Santavirta S, Konttinen YT, Saito T, Grönblad M, Partio E, Kemppinen P, et al. Immune response to polyglycolic acid implants. J Bone Joint Surg Br. 1990;72:597–600. doi: 10.1302/0301-620X.72B4.2166048 2166048

[36] Klagsbrun M. Large-scale preparation of chondrocytes. Methods Enzymol. 1979;58: 560–564. doi: 10.1016/s0076-6879(79)58171-3 423791

[37] Shasti M, Jacquet R, McClellan P, Yang J, Matsushima S, Isogai N, et al. Effects of FGF-2 and OP-1 in vitro on donor source cartilage for auricular reconstruction tissue engineering. Int J Pediatr Otorhinolaryngol. 2014;78: 416–422. doi: 10.1016/j.ijporl.2013.11.028 24439635

[38] Nishiwaki H, Fujita M, Yamauchi M, Isogai N, Tabata Y, Kusuhara H. A novel method to induce cartilage regeneration with cubic microcartilage. Cells Tissues Organs. 2017;204: 251–260. doi: 10.1159/000479790 28972948

[39] Liu CF, Samsa WE, Zhou G, Lefebvre V. Transcriptional control of chondrocyte specification and differentiation. Semin Cell Dev Biol. 2017;62: 34–49. doi: 10.1016/j.semcdb.2016.10.004 27771362 PMC5318237

[40] Liu CF, Lefebvre V. The transcription factors SOX9 and SOX5/SOX6 cooperate genome-wide through super-enhancers to drive chondrogenesis. Nucleic Acids Res. 2015;43: 8183–8203. doi: 10.1093/nar/gkv688 26150426 PMC4787819

[41] Malik A, Kanneganti TD. Function and regulation of IL-1α in inflammatory diseases and cancer. Immunol Rev. 2018;281: 124–137. doi: 10.1111/imr.12615 29247991 PMC5739076

[42] Tian F, Hosseinkhani H, Hosseinkhani M, Khademhosseini A, Yokoyama Y, Estrada GG, et al. Quantitative analysis of cell adhesion on aligned micro- and nanofibers. J Biomed Mater Res A. 2008;84: 291–299. doi: 10.1002/jbm.a.31304 17607759

[43] Neidel J, Zeidler U. Independent effects of interleukin 1 on proteoglycan synthesis and proteoglycan breakdown of bovine articular cartilage in vitro. Agents Actions. 1993;39: 82–90. doi: 10.1007/BF01975718 8285145

[44] Grinnell F. Cellular adhesiveness and extracellular substrata. In: Bourne GH, Danielli JF, editors. International Review of Cytology. Elsevier; Volume 53: 1978. pp. 65–144. 10.1016/s0074-7696(08)62241-x 208994

[45] Wright B, Parmar N, Bozec L, Aguayo SD, Day RM. A simple and robust method for pre-wetting poly(lactic-co-glycolic) acid microspheres. J Biomater Appl. 2015;30: 147–159. doi: 10.1177/0885328215577297 25791685 PMC4509882

[46] Dvořáková H, Čech J, Stupavská M, Prokeš L, Jurmanová J, Buršíková V, et al. Fast surface hydrophilization via atmospheric pressure plasma polymerization for biological and technical applications. Polymers (Basel). 2019;11: 1613. doi: 10.3390/polym11101613 31590313 PMC6836037

[47] Crowley C, Klanrit P, Butler CR, Varanou A, Platé M, Hynds RE, et al. Surface modification of a POSS-nanocomposite material to enhance cellular integration of a synthetic bioscaffold. Biomaterials. 2016;83: 283–293. doi: 10.1016/j.biomaterials.2016.01.005 26790147 PMC4762251

[48] Mikos AG, Lyman MD, Freed LE, Langer R. Wetting of poly(L-lactic acid) and poly(DL-lactic co-glycolic acid) foams for tissue culture. Biomaterials. 1994;15: 55–58. doi: 10.1016/0142-9612(94)90197-x 8161659

